# Supplementary material for: Middle east warming in spring enhances summer rainfall over Pakistan
Source: Nat Commun. 2023 Nov 22;14:7635. doi: 10.1038/s41467-023-43463-0 (PMC10665419; doi:10.1038/s41467-023-43463-0)
Supplement: Supplementary file 1 — Supplementary Information [file 41467_2023_43463_MOESM1_ESM.pdf]

Middle East Warming in Spring Enhances Summer Rainfall over Pakistan

Baosheng Li[1,2], Lei Zhou[2, 3*], Jianhuang Qin[2,4], Tianjun Zhou[5], Dake Chen[1,2,3], Shugui Hou[3], Raghu Murtugudde[6,7*]

1. *State Key Laboratory of Satellite Ocean Environment Dynamics, Second Institute of Oceanography, Ministry of Natural Resources, Hangzhou, China*
2. *Southern Marine Science and Engineering Guangdong Laboratory (Zhuhai), Zhuhai, China*
3. *School of Oceanography, Shanghai Jiao Tong University, Shanghai, China*
4. *College of Oceanography, Hohai University, Nanjing, China*
5. *State Key Laboratory of Numerical Modeling for Atmospheric Sciences and Geophysical Fluid Dynamics, Institute of Atmospheric Physics, Chinese Academy of Sciences, Beijing, China*
6. *Indian Institute of Technology Bombay, Mumbai, India*
7. *University of Maryland, College Park, Maryland, USA*

**Corresponding authors:*
Lei Zhou: zhoulei1588@sjtu.edu.cn
Raghu Murtugudde: mahatma@umd.edu

This PDF contains Supplementary Figures 1-7 and Supplementary Tables 1-2.

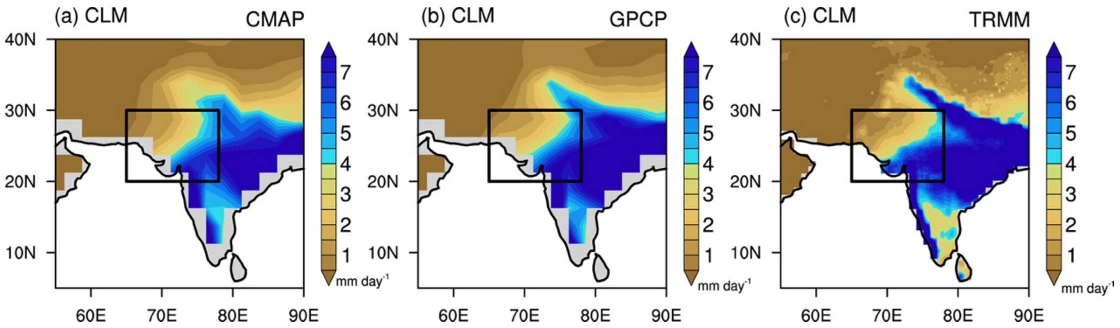

Supplementary Figure 1 | Climatological rainfall in summer. Mean rainfall over land (unit: mm day^{-1}) during JJAS for 1979-2022 based on (a) CMAP; (b) GPCP, and (c) TRMM datasets.

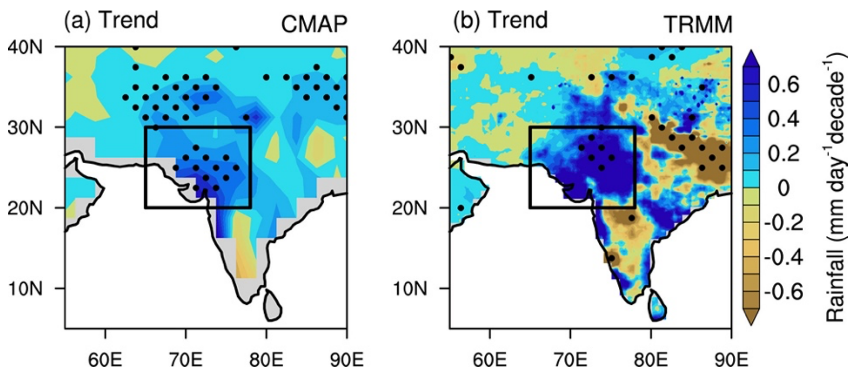

Supplementary Figure 2 | Summer rainfall increases over Pakistan. Trends in summer mean rainfall (unit: mm day^{-1} $decade^{-1}$) for 1979-2022 in (a) CMAP and (b) TRMM datasets. The dotted areas are significant at the 95% confidence level using the Mann-Kendall test.

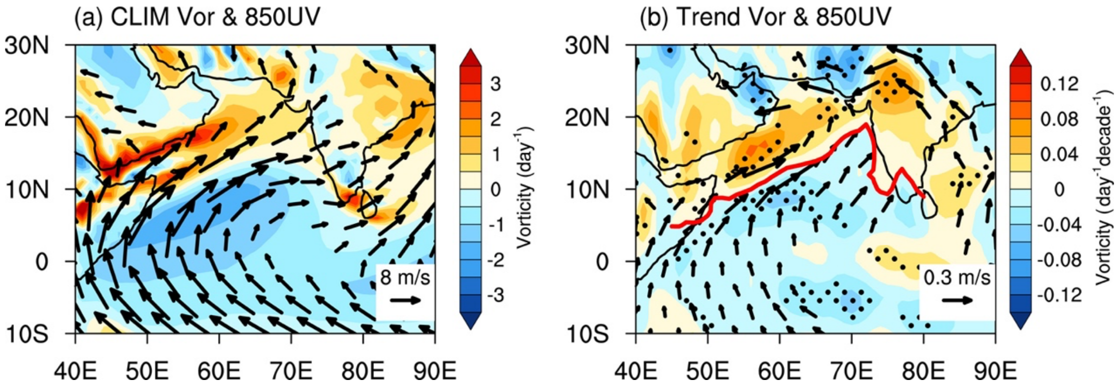

Supplementary Figure 3 | The climatology and trend of low-level jet in summer. (a) Mean absolute vorticity (shading; unit: day^{-1}) and mean horizontal winds at 850 hPa (vectors; unit: m s^{-1}) during JJAS for 1979-2022. (b) Trends in seasonal mean absolute vorticity (shading; unit: day^{-1} decade^{-1}) and in horizontal winds at 850 hPa (vectors; unit: m s^{-1} decade^{-1}) during JJAS for 1979-2022. The locations of zero absolute vorticity are marked by red contours, denoting the low-level jet maxima. The dotted areas are significant at the 95% confidence level using the Mann-Kendall test.

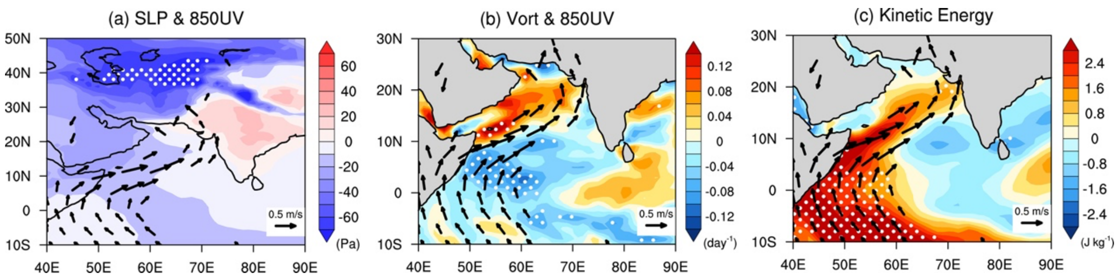

Supplementary Figure 4 | Dependence of low-level jet on Middle East heating. Regression coefficients of the (a) sea level pressure (SLP; shading; unit: Pa), (b) absolute vorticity (shading; unit: day^{-1}), (c) kinetic energy (shading; unit: J kg^{-1}) and horizontal winds at 850 hPa (vectors; unit: m s^{-1}) in early summer (May-June) onto the normalized land heating index in spring during the period of 1979-2022. The dotted areas are significant at the 95% confidence level using the Student's *t*-test. Only the wind anomalies that passed the 95% confidence level are shown.

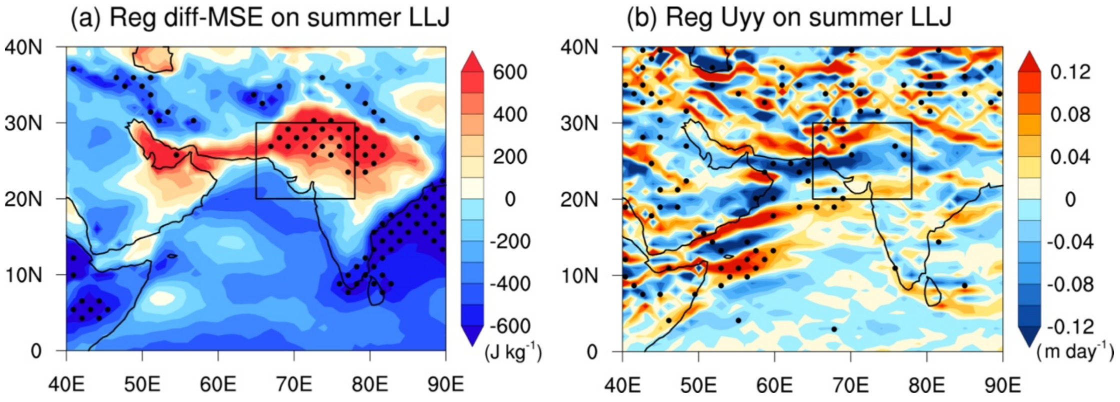

Supplementary Figure 5 | Dependence of dynamical instability on summer low-level jet. Regression coefficients of (a) the vertical difference of moist static energy (MSE) between 1000 and 500 hPa (units: J kg^{-1}), (b) the meridional gradient of quasi-geostrophic potential vorticity ($\beta - \frac{\partial^2 u}{\partial y^2}$, where β is the meridional gradient of Coriolis parameter and u is the zonal wind; units: m^{-1} day^{-1}) onto the normalized summer LLJ index during the period of 1979-2022. The dotted areas are significant at the 95% confidence level using the Student's *t*-test.

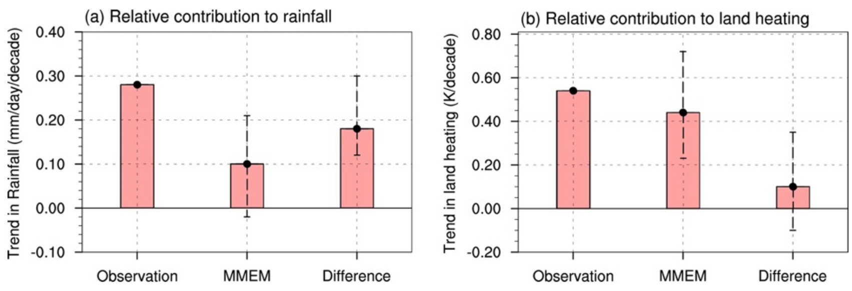

Supplementary Figure 6 | Relative contribution of external forcing and internal variability to Pakistan rainfall and Middle East land heating. Trends in (a) Pakistan summer rainfall (unit: mm day^{-1} decade^{-1}) and (b) spring skin temperature over the Middle East (unit: °C decade^{-1}) for 1979-2014. The bars with black dots indicate the average of trends in all model members, and the observations as certain values are obtained from the GPCP (rainfall) and the ERA5 (skin temperature). The upper and bottom dashed lines indicate the uncertainty of the trends in all 192 model members, which is calculated by one standard deviation of trends. The multi-model ensemble means (MMEM) denote the contribution of external forcing and the differences between the observation and the MMEM denote the contribution of internal variability.

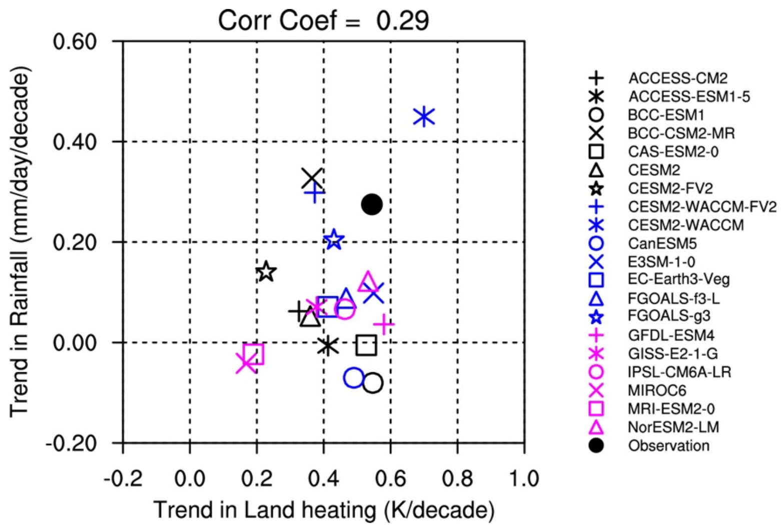

Supplementary Figure 7 | Simulation dependence of summer rainfall over Pakistan on Middle East heating in spring in CMIP6. Dependence of the model simulations between the trend in Pakistan rainfall in summer and the trend in Middle East land heating in spring. The x-axis denotes the trend in Middle East land heating in spring from 1979 to 2014. The y-axis is the trend in Pakistan's rainfall in summer from 1979 to 2014. All models from the CMIP6 are labeled by different markers. The observed rainfall is obtained from the GPCP and the observed land heating is obtained from the ERA5.

Supplementary Table 1 | Trend in the northward shift in LLJ (unit: ^{o} decade^{-1}) and Pakistan rainfall (mm day^{-1} decade^{-1}) under three scenarios. R^2 is the square of the correlation coefficient of the time series between LLJ movement and Pakistan rainfall. It denotes the contribution of LLJ shift to the rainfall changes.

	SSP2-4.5	SSP3-7.0	SSP5-8.5
LLJ movement	0.06	0.12	0.17
Pakistan rainfall	0.02	0.05	0.1
R^2	44%	59%	88%

Supplementary Table 2 | Institutions, horizontal resolutions for atmospheric components, and the number of realizations of the 20 models analyzed from the CMIP6. The bold are 11 models used to determine the future changes in horizontal winds and rainfall with SSP2-4.5, SSP3-7.0, and SSP5-8.5 provided.

Model Name	Resolution	Institution	Number of Realizations
**ACCESS-CM2**	144 x 192	Commonwealth Scientific and Industrial Research Organization (Australia)	2
ACCESS-ESM1-5	145 x 192		3
**BCC-CSM2-MR**	160 x 320	Beijing Climate Center (China)	3
BCC-ESM1	64 x 128		3
CAS-ESM2-0	128 x 256	Chinese Academy of Sciences (China)	50
CanESM5	64 x 128	Canadian Centre for Climate Modelling and Analysis (Canada)	4
**CESM2**	192 x 288	National Center for Atmospheric Research, Climate and Global Dynamics Laboratory (USA)	10
CESM2-FV2	96 x 144		3
**CESM2-WACCM**	192 x 288		3
CESM2-WACCM-FV2	96 x 144		3
E3SM1-0	180 x 360	E3SM-Project, Department of Energy (USA)	5
**EC-Earth3-Veg**	256 x 512	European Centre for Medium-Range Weather Forecasts	5
**FGOALS-f3L**	180 x 288	Institute of Atmospheric Physics, Chinese Academy of Sciences (China)	3
**FGOALS-g3**	80 x 180		3
**GFDL-ESM4**	180 x 288	National Oceanic and Atmospheric Administration, Geophysical Fluid Dynamics Laboratory (USA)	3
**GISS-E2-1-G**	90 x 144	Goddard Institute for Space Studies (USA)	38
**IPSL-CM6A-LR**	143 x 144	Institute Pierre-Simon Laplace (France)	32
MIROC6	128 x 256	Atmosphere and Ocean Research Institute, National Institute for Environmental Studies and Japan Agency for Marine-Earth Science and Technology (Japan)	10
MRI-ESM2-0	160 x 320	Meteorological Research Institute (Japan)	6
**NorESM2-LM**	96 x 144	Norwegian Climate Centre (Norway)	3
